# Effects of Information Overload, Communication Overload, and Inequality on Digital Distrust: A Cyber-Violence Behavior Mechanism

**DOI:** 10.3389/fpsyg.2021.643981

**Published:** 2021-04-20

**Authors:** Mingyue Fan, Yuchen Huang, Sikandar Ali Qalati, Syed Mir Muhammad Shah, Dragana Ostic, Zhengjia Pu

**Affiliations:** ^1^School of Management, Jiangsu University, Zhenjiang, China; ^2^Department of Business Administration, Sukkur Institute of Business Administration University, Sukkur, Pakistan; ^3^School of Finance and Economics, Jiangsu University, Zhenjiang, China

**Keywords:** cyber violence, information overload, communication overload, information inequality, digital distrust, negative emotions, SOR theory

## Abstract

In recent years, there has been an escalation in cases of cyber violence, which has had a chilling effect on users' behavior toward social media sites. This article explores the causes behind cyber violence and provides empirical data for developing means for effective prevention. Using elements of the stimulus–organism–response theory, we constructed a model of cyber-violence behavior. A closed-ended questionnaire was administered to collect data through an online survey, which results in 531 valid responses. A proposed model was tested using partial least squares structural equation modeling using SmartPLS 3.0, v (3.2.8). Research findings show that information inequality is a strong external stimulus with a significant positive impact on digital distrust and negative emotion. However, the effects of information overload on digital distrust and the adverse effects of communication overload on negative emotions should not be ignored. Both digital distrust and negative emotions have significant positive impacts on cyber violence and cumulatively represent 11.5% changes in cyber violence. Furthermore, information overload, communication overload, information inequality, and digital distrust show a 27.1% change in negative emotions. This study also presents evidence for competitive mediation of digital distrust by information overload, information inequality, and cyber violence. The results of this study have implications for individual practitioners and scholars, for organizations, and at the governmental level regarding cyber-violence behavior. To test our hypotheses, we have constructed an empirical, multidimensional model, including the role of specific mediators in creating relationships.

## Introduction

Cyber violence refers to any behavior on the Internet advocating violence or using language calculated to inflame the passions to achieve mass emotional catharsis (Hou and Li, 2017), which can be considered an extension of social violence to the Internet (Li et al., 2017). In the past 2 years, with the rapid growth of Internet users, cyber violence incidents on social media have appeared frequently, including such behavior as bullying, flaming, and verbal abuse, and even death threats. For example, Doctor An, the protagonist of the “collision” incident at the Deyang swimming pool in 2018, committed suicide as a result of the added stress of cyber violence. During the COVID-19 (coronavirus disease 2019) epidemic in 2020, private information such as ID numbers and photographs of confirmed COVID-19 patients and people who live in Hubei province was widely spread on social networks. This resulted in suffering and serious secondary harm to the parties involved from excessive hardcore prevention and control. Studies have shown that cyber violence can have serious adverse consequences on an individual's psychology and physiology (Sincek et al., 2017; Backe et al., 2018) and is a major factor in fomenting social instability. Therefore, clarifying the mechanism of cyber violence and understanding its essence constitute a worthwhile goal for governmental policy makers to develop scientific guidelines for relevant online behavior, as well as protecting the physical and mental health of Internet users and maintaining social stability and unity.

In the academic world, domestic and foreign scholars from many fields, such as political philosophy (Finlay, 2018), law (Cheung, 2009), media (Zhang, 2012), sociology (Owen et al., 2017), and psychology (Hou and Li, 2017), have made many useful contributions to the study of cyber violence. The research concerns of Chinese scholars with regard to cyber violence mainly focus on analyzing the type of behavior and key influences, the characteristics of the current situation, and new development trends in governance. The research methods mainly involve qualitative analysis and typical case studies. These investigations have produced a wealth of data, providing a solid theoretical basis and practical information for future scholars. However, the work to date has mainly centered on the occurrence and development of cyber violence from the macro and medium perspective. Only rarely have investigators combed through the antecedents and internal mechanisms of the formation of cyber violence from the viewpoint of individual users. Therefore, utilizing the theoretical framework of stimulus–organism–response (SOR) modeling, this article combines external environmental stimuli with individual cognition to explore the relationships between the various factors that generate cyber violence to reveal its mechanism.

## Theoretical Background and Hypotheses Development

### Theoretical Background

In the 1970s, some scholars began to realize that all aspects of the environment are external stimuli that affect an individual's cognition and emotion, which in turn influences them toward responsible behavior, leading to the proposal of the well-known SOR theory (Mehrabian and Russell, 1974). Domestic scholars have applied SOR theory to the study of consumers' shopping behavior (Zhang and Lin, 2017), online shopping trends (Syastra and Wangdra, 2018), employee complaints (Xie et al., 2019), and many other social phenomena. Thus, SOR theory and its associated framework have become accepted as the primary research tools for effectively analyzing individual behavior. Based on previous studies and SOR theory, this article holds that the formation of cyber violence progresses through three stages, namely, influences of environmental stimuli (S), psychological activity of the organism (O), and its collective responses (R). Our model has the following attributes:

1. In terms of external stimulation, individual users are affected by the information and communication overload (CO) unique to the digital era, and the information inequality (II) caused by the information cocoon room effect.
2. The combined effects of the above stimuli cause individuals to have complex psychological reactions, such as feeling of doubt and disagreement among users, expressed as digital distrust (DD) and negative emotions such as anxiety and disgust.
3. Individual reactions may take the form of specific negative behavior, such as cyber violence.

Based on the above analysis, behavior leading to cyber violence is essentially a kind of feedback from digital users for coping with negative influences from the external environment combined with internal psychological stress. The external environmental factors include information overload (IO), CO, and II. Online distrust and negative emotions are typical psychological responses to these external factors. Cyber violence is the behavioral feedback resulting from these individual psychological activities.

### Hypotheses Development

#### Information Overload

IO refers to the situation where information exceeds the ability of a user to process and utilize it, resulting in negative feelings of failure. IO is a function of information quality and quantity—specifically, excessive quantity and poor quality (Rong, 2010; Cheng et al., 2014). Excess information is usually a result of redundancy, and users can reduce this factor with various digital technologies that continuously compare the effectiveness of information among users and increase the possibility of disagreements among users. The decrease in information quality makes users doubt the truth of the information presented and have to take time to determine its veracity. When users have to spend too much time in an attempt to obtain effective information, negative emotions will occur (Asif Naveed and Anwar, 2020). The research of Congard and Carole (2020) on IO in the international network environment also showed that too much information will generate negative emotions among users. Therefore, this article proposes the following hypotheses:

*H1a:* IO has a significant positive effect on DD.

*H1b:* IO has a significant positive effect on negative emotions.

#### Communication Overload

CO refers to a situation in which a network's communication needs exceed an individual's communication ability (Cho et al., 2011; Tripathy et al., 2016), which can interrupt a user's study or work schedule (Cao and Sun, 2017). CO can disturb the normal routine of users (Mcfarlane and Latorella, 2002), and the frequent interruptions make it difficult for them to concentrate (O'Connail and Frohlich, 1995; Mcfarlane, 1998). This can lead to a decline in the accuracy of judgment and thus negatively affect users' feelings about trusting the information and even other individuals (Mcfarlane and Latorella, 2002). At the same time, when faced with social communications that have to be dealt with, users who lack effective communication skills may be at a loss and can suffer from fatigue and anxiety (Mcfarlane, 1998). Consequently, we proposed the following hypotheses:

*H2a:* CO has a significant positive effect on DD.

*H2b:* CO has a significant positive effect on negative emotions.

#### Information Inequality

II results from inherent social inequities that affect how people at different socioeconomic levels gain access to information and what types of information are distributed to them. Differences in information access are mainly related to the level of technological development in a region, but also to individual educational background. Good education and high-quality social resources generally ensure equal access to and use of information technology and information resources (Figueiredo, 2018). The essence of information distribution inequality is the selectiveness inherent in the power to control information, which is considered a necessary element of resource distribution (Hargittai and Hsieh, 2013). As inequities in information access have been largely addressed by most countries, the II discussed in this article will mostly refer to its unequal distribution. Controlling who gets what information is much easier on Internet-based platforms than with print media. Mastering the technology for channeling fragmented information according to some algorithm has allowed media giants to take advantage of the system to restrict distribution and deprive users of their right to information. II selectively influences users' perceptions, reduces comprehension and objective understanding of the world, and lowers trust among users. When information flow is selectively altered to make it more homogeneous, the user's rational and cognitive abilities become inoperable. Rational thinking cannot take place when facts are withheld or misrepresented. In such a situation, differences of opinion are increased, and communication barriers occur among different groups, resulting in further narrowing of information bandwidth and the creation of an information “cocoon” around the users, producing cognitive dissonance in the group and increased communication difficulties (Hargittai and Hsieh, 2013). When the differences in values between groups are constantly expanding, negative emotions are likely to occur. To address this, we proposed the following hypotheses:

*H3a:* II has a significant positive impact on DD.

*H3b:* II has a significant positive effect on negative emotions.

#### Digital Distrust

The Pew Research Center released a study entitled “The Future of Well-Being in a Tech-Saturated World” in April 2018 (Janna and Lee, 2018). In the report, DD was defined for the first time as exclusion among digital technology users. When people believe that others are better than themselves, DD will reduce individual initiative and intensify the further weaponization of shock, fear, anger, humiliation, and other emotions on the Internet, thus causing disagreement and questioning (Judith, 2016). People's positive emotion is the mental state represented as “trust,” whereas the negative emotion is the mental state represented by “distrust” (Sha et al., 2015). DD can divide users, create negative emotions, and potentially tear society apart, making negative behavior such as cyber violence more likely to occur. Consequently, the following hypotheses were proposed:

*H4:* DD has a significant positive effect on negative emotions.

*H5:* DD has a significant positive effect on cyber violence.

#### Negative Emotions

Negative stimuli can produce negative emotions (Liu and Liu, 2013). In the Internet environment, users' negative emotions include regret, anxiety, fear, disgust, irritability, etc. (Ruensuk et al., 2019). In his research on China's cyber violence from the perspective of initiators and participants, Hou and Li (2017) showed that the motivations for cyber violence could be divided into moral judgments and cathartic malicious attacks, arising from cathartic emotions. The Internet provides a convenient place for people to vent their emotions, but if this is done carelessly, it can result in the rapid spread of negative feelings and the instigation of cyber violence. Therefore, the following hypothesis is proposed:

*H6:* Negative emotions have a significant positive effect on cyber violence.

#### The Mediating Role of Digital Distrust

For products that they have purchased, consumers often post comments or reviews in which they rate the item positively or negatively according to their experience with it. The negative deviation theory states that negative information can have a powerful deterrent effect on users, in contrast to positive or neutral information. Other researchers (Zhu et al., 2020) found that positive information was considered more trustworthy than negative information. Furner and Zinko (2017) studied the influence of IO on the development of trust and purchase intention based on online product reviews in a mobile vs. web context. The results confirmed that IO had a strong influence on trust. Furner Christopher et al. (2016) revealed an association between IO, trust, and purchase intention. A recent study (Zhu et al., 2020) supported the idea of a mediating role of trust and satisfaction between information quality, social presence, and purchase intention. Therefore, this article proposed the following hypothesis:

*H7:* DD mediates the relationship between information and CO and II.

**Figure 1:**
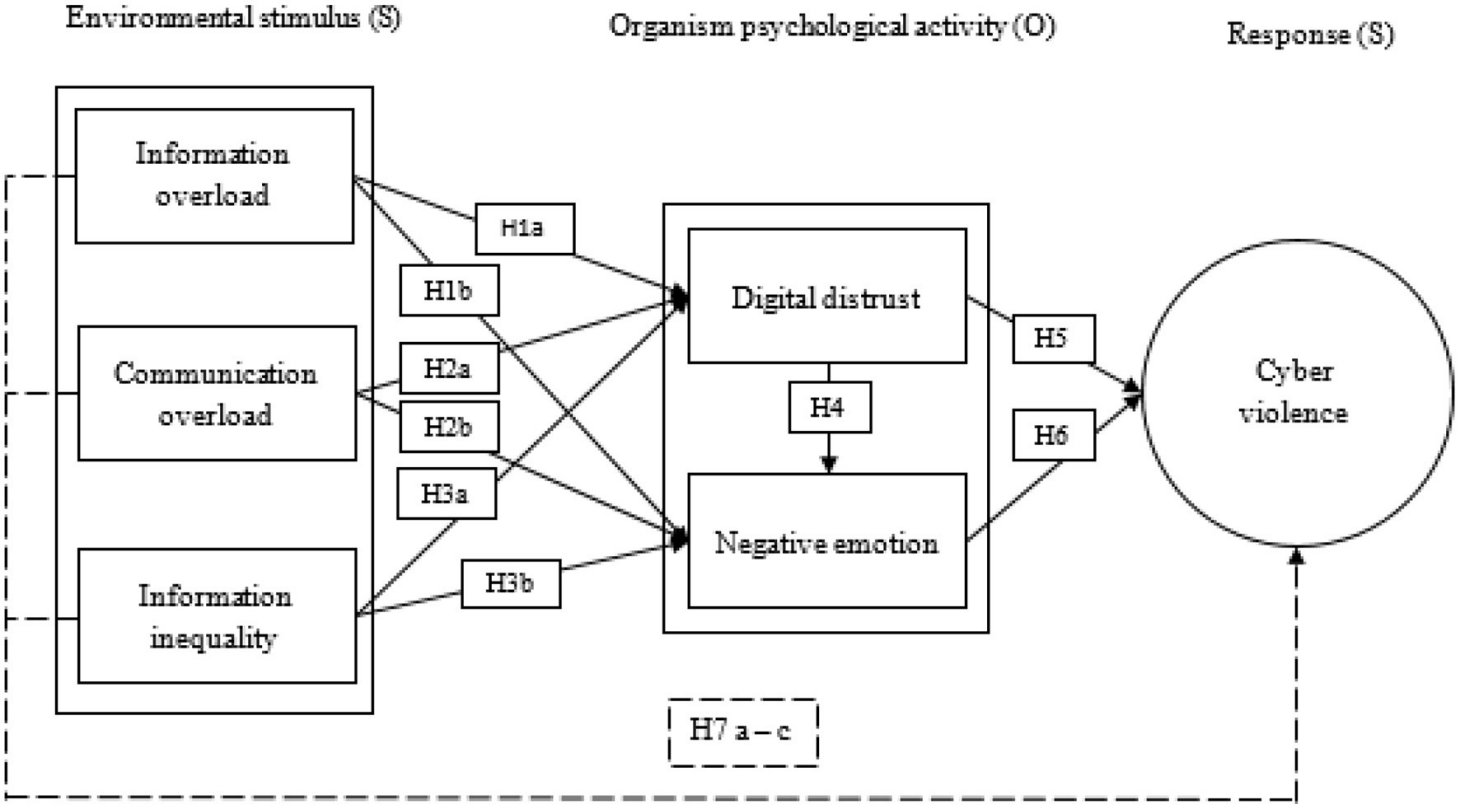
Theoretical model.

Our SOR-based cyber violence mechanism model constructed according to the above assumptions is shown in Figure 1.

## Research Methodology

### Sample and Procedure

A sample of 531 was obtained from general public using a convenient random sampling approach because of time and budget constraints. A closed-ended questionnaire was administered by email and through online platforms such as WeChat and WhatsApp. Online surveys are often used when the population is large (Tian et al., 2020). Authenticated online surveys are considered a valid tool for new research and provide a fast, simple, and less costly approach to collecting data (Qalati et al., 2021). The formal survey was designed and conducted from February 2020 to April 2020, and the major reason for collecting data in 4 months' lag time was to mitigate common method bias (CMB) (Li et al., 2020). In the present study, 783 questionnaires were collected from a general audience, of which 252 were invalidated because of a selection of the same option and the use of the same IP for the response. This left 531 valid questionnaires for an effective response rate of 67.82%.

### Respondents' Information

Table 1 reflects that a total of 322 male and 209 female participants accounted for 60% and 40%, respectively. Respondents younger than 25 (245), 26–30 (146), 31–40 (67), 41–50 (41), and older than 50 years accounted for 46.1, 27.5, 12.7, 7.7, and 6%, respectively. Regarding the frequency of microblog usage, approximately more than one-third of participants accounted for once a day (35.6%) and several times a day (36.9%), whereas more than one-quarter of them (27.5%) for several times a week. Regarding the duration of microblog usage under a year (42), 1–2 (151), 2–3 (157), 3–4 (67), and more than 4 (114) years accounted for 7.9, 28.4, 29.6, 12.6, and 21.5%, respectively.

**Table 1:** Descriptive statistics.

**Variable**	**Type**	**No. of people**	**Proportion**
Gender	Male	322	60.60%
	Female	209	39.40%
Age (years)	<25	245	46.10%
	26–30	146	27.50%
	31–40	67	12.70%
	41–50	41	7.70%
	>50	32	6.00%
Frequency of	Several times a day	196	36.90%
microblog usage	Once a day	189	35.60%
	Several times a week	146	27.5%
Duration of	<1	42	7.90%
microblog usage	1–2	151	28.40%
(years)	2–3	157	29.60%
	3–4	67	12.60%
	>4	114	21.50%

### Measures

The study used seven-point Likert scales (1 = very unimportant to 7 = very important) to record responses. A pilot survey containing 21 questions was first conducted to validate the questionnaire method. After analyzing and discussing the pilot study results with experts, the questions, wording, and semantics of the questionnaire were modified, resulting in a final list of 18 items that were issued as the formal questionnaire (**Table 3**). IO was measured using three items from Karr-Wisniewski and Lu (2010), Lee et al. (2016). CO was assessed using three items (Cho et al., 2011; Lee et al., 2016; Shi et al., 2020). II was measured using three items adapted from Lee et al. (2016) and Yu et al. (2017). DD was measured using three items from Zhu et al. (2020). Negative emotions were assessed using three items adapted from Kang et al. (2013) and Chiu et al. (2020), and cyber violence was assessed using three items from Lee et al. (2016) and Sari and Camadan (2016).

### Data Analysis

To consider the influence of multiple variables on the model and verify the validity of the theoretical hypotheses, structural equation modeling (SEM) was used to analyze the data. SPSS 24.0 and SmartPLS 3.2.8 were the statistical software used in the study. In particular, SPSS was used for descriptive information of the participants and some test related to CMB and sample adequacy, whereas SmartPLS 3.0 was used for partial least squares (PLS) SEM because it is widely used across fields (Hair Joseph et al., 2019; Ahmed et al., 2020). Furthermore, it is considered a comprehensive software program with an intuitive graphical user interface to run PLS-SEM analysis, which certainly has had a massive impact. Besides, it enables the specification of complex interrelationships between observed and latent constructs (Hair Joseph et al., 2019).

## Results

### Common Method Bias and Bartlett Spherical Test

The Kaiser–Meyer–Olkin (KMO) value and Bartlett spherical test value of sample data were calculated. The KMO value was 0.745, the chi-squared value was 4,199.094, the degrees of freedom were 276, and the *p* < 0.001 (Table 2). The larger the KMO value, the stronger the correlation between variables. The variables were suitable for factor analysis, and the structural validity between variables was good (Chen et al., 2016). This study used three approaches to detect the CMB. First, Harman's single factor test stated that the first factor represents only 16.18% of the variance, which is far below the acceptable threshold (50.0%) (Podsakoff et al., 2003). Second, variance inflation factor (VIF), which is called the full-collinearity approach using SmartPLS (Ali Qalati et al., 2021), requires to be ≤ 3 (Hair Joseph et al., 2019) (Table 3). Third, Bagozzi et al. (1991) proposed that if the correlation among the constructs was >0.9, there is evidence of CMB. However, none of the constructs was found to be greater than the minimum cutoff value (Table 4).

**Table 2:** KMO and Bartlett test.

**Kaiser–Meyer–Olkin measure of sampling adequacy**.		**0.745**
Bartlett test of sphericity	~χ^2^	4,199.094
	*df*	276
	Sig.	0.000

**Table 3:** Measurement model.

**Variables**	**Item**	**Loadings**	**CA**	**CR**	**AVE**	**VIF**
Communication overload	CO1: I often receive more information than I can handle	0.822	0.843	0.902	0.754	1.013
	CO2: I often send more messages than I expect	0.887				
	CO3: Too many messages and notifications have been interrupting my daily life at the moment	0.893				
Cyber violence	CV1: I would vilify others on the Internet	0.911	0.857	0.912	0.776	
	CV2: I will disclose other people's privacy on the Internet	0.888				
	CV3: I would threaten people on the Internet	0.841				
Digital distrust	DD1: The information pushed to me by the Internet makes me question it	0.891	0.815	0.887	0.723	1.044
	DD2: I worry about other people getting information that's more useful than mine	0.831				
	DD3: I have a different view of the same information being pushed online than others	0.827				
Information inequality	II1: I don't think I get the same information as other people on the Internet	0.887	0.823	0.894	0.738	1.025
	II2: The amount of information the Internet pushes to me and to others is inconsistent	0.894				
	II3: The quality of information the Internet pushes to me and to others is inconsistent	0.793				
Information overload	IO1: I think the Internet gives me too much information	0.922	0.782	0.863	0.679	1.023
	IO2: I don't think the quality of information on the Internet is very high these days	0.76				
	IO3: I feel that too much information of low quality prevents me from getting good information	0.78				
Negative emotion	NE1: I'm getting less information online than I used to	0.801	0.786	0.875	0.7	1.019
	NE2: Frequent use of the Internet makes me tired	0.86				
	NE3: Now I hate the information pushed by the Internet	0.848				

**Table 4:** Discriminant validity.

**Variables**	**1**	**2**	**3**	**4**	**5**	**6**
Communication overload	0.868					
Cyber violence	−0.065	0.881				
Digital distrust	−0.069	0.308	0.85			
Information inequality	−0.026	0.169	0.156	0.859		
Information overload	−0.097	0.059	0.121	0.009	0.824	
Negative emotion	−0.096	0.18	0.137	0.51	−0.006	0.837

### Measurement Model

According to Roldán and Sánchez-Franco (2012), a proposition to measure the model is required to assess the individual items for reliability, internal consistency, content validity, convergent validity, and discriminant validity. Individual item reliability was measured by outer loadings of items related to a particular dimension (Hair et al., 2012). Hair et al. (2016) recommended that factor loading should be between 0.40 and 0.70, whereas Hair et al. (2017) proposed a value of ≥0.7 (Table 3). According to Nunnally (1978), Cronbach α values should exceed 0.7: the threshold values of constructs in this study ranged between 0.782 and 0.857. The internal consistency reliability (Bagozzi and Yi, 1988) required that the composite reliability (CR) be ≥0.7, and the CR coefficient values in this study were between 0.863 and 0.912. Regarding convergent validity, Fornell and Larcker (1981) recommended that the average variance extracted (AVE) should be ≥0.5. The AVE values in this study were between 0.679 and 0.776, confirming a satisfactory level of convergent validity. With regard to discriminant validity, Fornell and Larcker (1981) stated that the square root of the AVE for each construct should exceed the correlation of the construct with other model constructs. Table 4 gives the discriminant validity of the results.

### Structural Model

**Figure 2:**
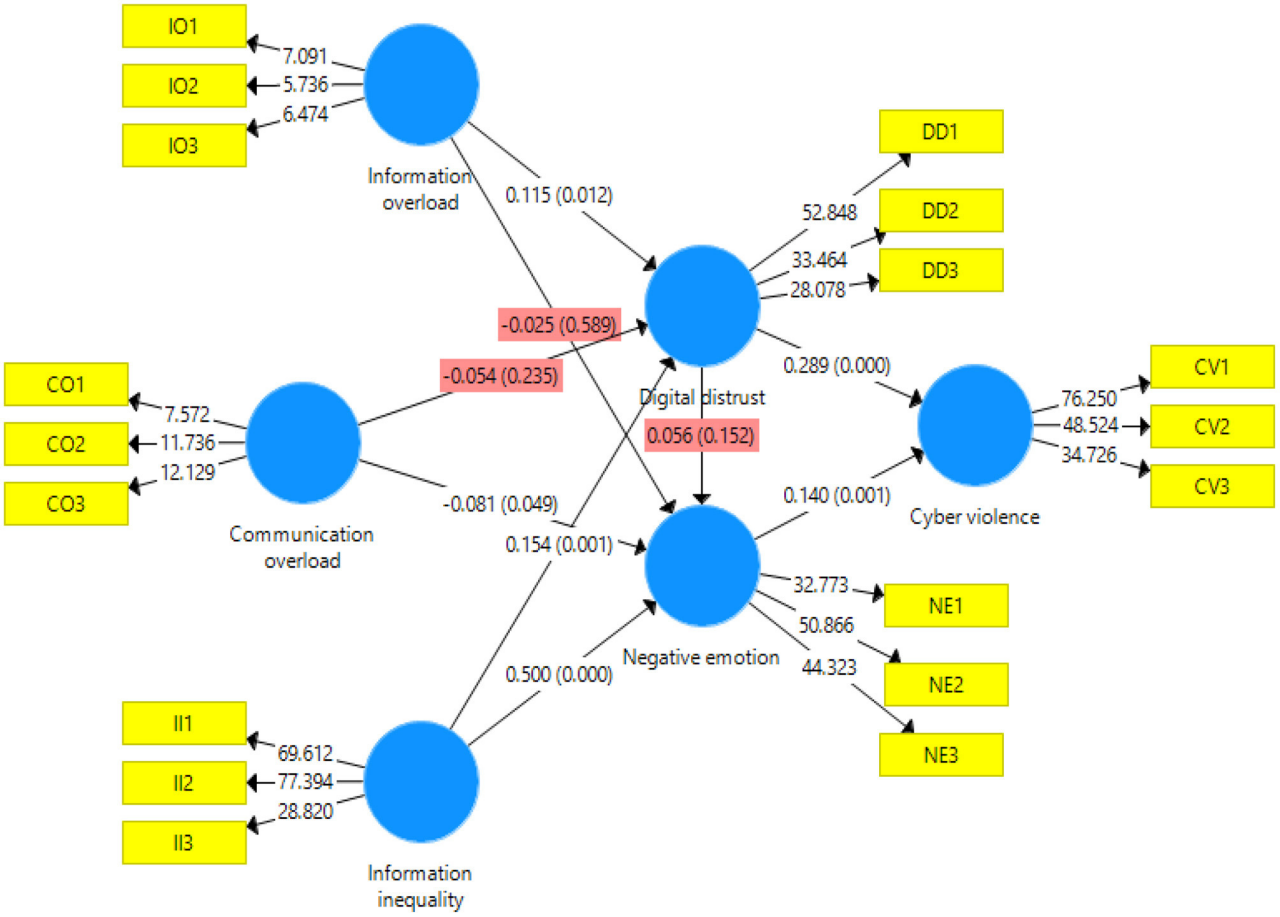
Structural equation modeling.

In this article, SmartPLS 3.2.8 was used to estimate the path coefficient and verify the hypothesis. This article used PLS bootstrapping with 5,000 bootstraps for the 531 cases to demonstrate results associated with the path coefficients and their significance level (Table 5). Among them, H1a, H2b, H3a, H3b, H5, H6, H7a, and H7c passed the significance test. However, the path coefficient of H2b was −0.081, which proves the hypothesis stating that CO had a significant negative impact on negative emotions, but it is very close to the rejection cutoff. The model path analysis found that the hypotheses, H1b, H2a, H4, and H7b, were not valid. The graphical presentation of the model with path and significance level is shown in Figure 2. The results can be interpreted to conclude that IO and II have a significant positive impact on DD and that II has a significant positive effect on negative emotions. CO has a significant negative impact on negative emotions. DD and negative emotions have a significant positive effect on cyber violence. According to Zhao et al. (2010), there are five types of mediation: complementary, competitive, indirect only, direct only, and no effect (non-mediated). The authors stated that if the mediated effect (*a* × *b*) and direct effect *c* both exist and point in the same direction, it is called complementary mediation. The evidence from this study proves that DD is a complementary mediator. There is no global measure of goodness of fit in SmartPLS-SEM (Hair et al., 2011).

**Table 5:** Path coefficients and hypothesis testing.

**Hypothesis**	**Relationship**	**Path coefficient**	**SD**	***t*-value**	**Decision**
**Direct effect**					
H1a	Information overload → digital distrust	0.115	0.046	2.514^*^	Supported
H1b	Information overload → negative emotion	−0.025	0.047	0.541	Not supported
H2a	Communication overload → digital distrust	−0.054	0.046	1.186	Not supported
H2b	Communication overload → negative emotion	−0.081	0.041	1.968^*^	Supported
H3a	Information inequality → digital distrust	0.154	0.045	3.394^***^	Supported
H3b	Information inequality → negative emotion	0.500	0.043	11.602^***^	Supported
H4	Digital distrust → negative emotion	0.056	0.039	1.434	Not supported
H5	Digital distrust → cyber violence	0.289	0.045	6.37^***^	Supported
H6	Negative emotion → cyber violence	0.140	0.043	3.272^***^	Supported
**Indirect effect**					
H7a	Information overload → digital distrust → cyber violence	0.033	0.015	2.276^*^	Supported
H7b	Communication overload → digital distrust → cyber violence	−0.016	0.014	1.137	Not supported
H7c	Information inequality → digital distrust → cyber violence	0.045	0.015	2.938^*^	Supported

Path coefficient significance level

* P < 0.05,

** P < 0.01,

*** *P < 0.001*.

According to Cohen (1988), *R*^2^ values of 0.60, 0.33, and 0.19 are, respectively, substantial, moderate, and weak. However, Falk and Miller (1992) argued that an *R*^2^ value as low as 0.10 is also acceptable. The *R*^2^ value for this study was 0.115 for cyber violence. This suggests that DD and negative emotion explain 11.5% of the variation (Table 6), as the *R*^2^-value is sufficiently above the minimum cutoff according to Falk and Miller (1992). This study also employed the cross-validated redundancy measure (*Q*^2^) to evaluate the model. Henseler et al. (2009) suggested that a *Q*^2^ > 0 shows that the model has predictive relevance. Values of 0.02, 0.15, and 0.35, respectively, indicate that an exogenous construct has a small, medium, or considerable predictive relevance for a specific endogenous construct. The current study's model has weak predictive relevance for cyber violence and DD and medium for negative emotions (Table 6).

**Table 6:** Strength of the model.

	**Cross-validated redundancy**			**Coefficient of determination**	
**Construct**	**SSO**	**SSE**	***Q*^2^ (=1–SSE/SSO)**	***R*^2^**	**Adjusted *R*^2^**
Cyber violence	1,593	1,464.73	0.081	0.115	0.111
Digital distrust	1,593	1,555.96	0.023	0.042	0.036
Negative emotion	1,593	1,313	0.176	0.271	0.265

*SRMR, 0.054; d_ULS, 0.494; d_G, 0.260; χ^2^, 874.143*.

Standardized root mean square residual (SRMR) was used to assess the goodness of fit. SRMR is an absolute measure of fit: a value of zero indicates a perfect fit, and a value of <0.08 is considered a good fit (Hu and Bentler, 1999). In this study, the SRMR value obtained was 0.054, which is well-below the minimum cutoff (Table 6).

## Discussion

Utilizing the results from 531 valid questionnaires, five of the nine hypotheses regarding the mechanism of cyber violence in this article were verified, and all of them were significantly correlated. IO and II have a significant positive impact on DD, as supported by hypotheses H1a and H3a. Thus, digital users need to sift through the overload of information in order to dispel doubts and prevent disagreements with others. Because the user has gradually lost control of information access, effective information acquisition is more difficult. This results in a growing divergence between users that eventually creates DD. There are two main reasons for this. On the one hand, it is one of the characteristics of the digital age that information redundancy and information quality decline when IO exists. On the other hand, digital users have come to see that technological empowerment in the digital era has resulted in information control. This may manifest as problems such as living in an information cocoon and being subject to algorithm discrimination, which prevents users from obtaining the full complement of information and using it for production and creation. This conclusion is consistent with previous studies. II (path coefficient = 0.149) has a slightly stronger effect on DD than IO (path coefficient = 0.114). This demonstrates that the control of information by businesses empowered by digital technology has emerged as a growing threat to online shoppers and social media users. Therefore, improving information quality, reducing redundancy, and strengthening users' rights to access all information will help to reduce DD.

II has a significant positive impact on negative emotions, as supported by our findings in support of H3b. The more that digital users feel the unequal power of information control, the stronger their negative emotions will be. This conclusion is consistent with previous studies. The development of a digital society has led users to pay more attention to the value of information. Possessing excellent opportunities for searching, processing, and analyzing information is the sine qua non for success in social competition. However, when information access and distribution are deliberately manipulated over a period of time, conflicts among social groups will grow, and their mutual interests will diverge to the point where the different social groups may be torn apart, and severe negative social emotions will be the result. To prevent this from happening, stricter limits on information control by businesses need to be enacted and enforced. Internet users should be given equal access to and use of information to reduce differences of opinion and communication barriers between different groups to eliminate negative emotions.

DD and negative emotions have a significant positive impact on cyber violence, supported by findings from the H5 and H6 paths. The more serious the doubts and differences among digital users, the stronger will be the associated negative emotions, and the more serious the online violence. Compared with negative emotions (path coefficient = 0.176), DD has a higher degree of influence (path coefficient = 0.322), which indicates that the causes of cyber violence are not only the negative emotions associated with the emotional catharsis of users, but the doubts and disagreements between users that have become embedded in their minds. The goal, then, is the construction of a free and fair communication channel between users, eliminating doubts and disagreements, providing a clean and honest Internet environment, to stop negative emotions and cyber violence.

CO negatively affects negative emotions but is inconsistent with the H2b hypothesis, so the path is not supported. This suggests that digital users believe that negative emotions and behavior do not increase even in the presence of unmanageable communication and IO. This conclusion is inconsistent with previous studies, which may be due to the fact that CO has been widely perceived as an emerging social problem. However, compared with the more prominent problematic Internet use or social network addiction, it appears to have a lower impact and is easily ignored (Gui and Büchi, 2019). In addition, academic research results on the correlation between CO and negative emotions are inconsistent. For example, Chen and Lee concluded that CO caused by too frequent use of Facebook produced anxiety (Chen and Lee, 2013), whereas Jelenchick et al. found that the constant use of this social network on the university campus had nothing to do with anxiety (Jelenchick et al., 2013). Therefore, the conclusions drawn from this article should be tempered by the consideration that Chinese digital users may not have a systematic understanding of CO, and the results may be related to the type of Internet platform and study group, as well as their environment and cultural background.

In this article, H1b, H2a, H4, and H7b were not verified; however, that does not mean that IO has no effect on negative emotions or that CO does not foster DD. DD had no effect on negative emotions and no mediating effect between CO and cyber violence. There are many possible reasons for the lack of verification of the hypotheses, but these will have to be pursued in subsequent studies. The conclusions from this investigation are that to circumvent the generation of cyber violence based on the perspective of individual users, more attention should be paid to the influence of IO, II, DD, and negative emotions, as well as their significant correlation.

### Theoretical Implications

This study has several theoretical implications and makes a significant contribution to the existing literature on cyber violence. First, prior studies have paid more attention to information quality and less to the cumulative effects of IO, CO, and II. Second, this study leverages the application of the SOR model to the online environment, where the responses can be treated as new stimuli. Third, this study is grounded on the SOR theory to explain the intrinsic association between information, communication, II, and DD, negative emotions, and cyber-violence behavior. It should be noted that CO is not a significant antecedent of either DD or negative emotion, whereas IO and inequality comprise significant drivers of both. Our findings highlight the important mediating role of DD and negative emotions, revealing the clear association between an individual's perceptions of the digital world and its power to generate negative emotions. Therefore, this study can be considered as a new approach to understanding the path from stimuli such as IO, CO, and II to Internet behavior mediated by DD and negative emotions in the context of e-commerce.

### Practical Implications at the National, Enterprise, and Individual Level

At the national level, the government and relevant departments should impose new laws and regulations to clearly define the boundaries of information rights and interests between enterprises and users and to ensure that all users share equally in the access to information. The government need to be more proactive in regulating the network environment, establishing a spam tracing mechanism, punishments for deliberate misinformation, and controls on the generation and dissemination of low-quality, redundant information. Digital users should be encouraged to communicate with the authorities about incipient problems before they become a source of differences and doubts. Purging social groups of negative emotions necessitates the creation of a positive Internet ecosystem.

At the enterprise level, emerging Internet businesses need to establish the survival logic of “maximization of users' rights and interests rather than profit maximization.” Managers need to reconfigure the enterprise's value system and ease up on the control of information access and distribution to restore users' information rights and interests. The corporate Internet information review system needs to replace the production and diffusion of useless information with diversified high-quality information, to help users understand the world and reach a social consensus. More attention should be paid to users' emotional needs, and the engendering of negative emotions by the media should be stopped.

At the individual level, users should maintain a positive and optimistic attitude, but still be alert to the formation of information cocoons and algorithmic discrimination. They should take steps to enhance their information literacy, effectively identify and filter out low-quality information, and cultivate their information search and analysis ability. Their goals should be to strengthen their level of self-cognition, objectively view the self's need for information, conscientiously obtain objective information from multiple channels and points of view, and strive to reduce doubts and disagreements.

The theoretical model developed in this article is applicable to the analysis of the mechanism of cyber violence and provides the basis for a reasonable explanation of the formation of DD and negative emotions, which can be further explored in future studies.

### Limitations and Future Research Suggestions

This study has certain limitations that highlight avenues for future research. We used a convenient random sampling approach because of budget and time constraints. Collecting data from China only limits our findings' generalizability, and future studies should employ a cross-cultural approach to investigate the causes of cyber-violence behavior. As data were collected through an online survey, which also impedes the generalizability of findings in other countries, thus we call for future studies to include field surveys. The questionnaire survey scope could be expanded to increase the accuracy of the research results, as the sample size and reach may also be considered a limitation. Future studies could also investigate the effects of the government's policy on the mechanism.

The selection of research variables could be further subdivided into the internal environment and external environment variables to probe more deeply into the etiology of network violence. The follow-up will continue to analyze the above issues in detail to provide more theoretical and practical guidance for research on cyber violence.

Comparative studies could also be performed to validate our results and expand application of the proposed model using organizations and small- and medium-sized enterprises. This study did not examine the relationship between participants' attitudes, DD, negative emotions, and intentions, and future programs should investigate the influence of participant's attitudes and intentions. Finally, the mediating role of DD and negative emotions should be explored further.

## Data Availability Statement

The original contributions presented in the study are included in the article/supplementary material, further inquiries can be directed to the corresponding author/s.

## Ethics Statement

The studies involving human participants were reviewed and approved by Jiangsu University. The patients/participants provided their written informed consent to participate in this study.

## Author Contributions

All authors listed have made a substantial, direct and intellectual contribution to the work, and approved it for publication.

## Conflict of Interest

The authors declare that the research was conducted in the absence of any commercial or financial relationships that could be construed as a potential conflict of interest.
